# In vitro evaluation of the synergistic antioxidant and anti-inflammatory activities of the combined extracts from Malaysian *Ganoderma lucidum* and Egyptian *Chlorella vulgaris*

**DOI:** 10.1186/s12906-018-2218-5

**Published:** 2018-05-10

**Authors:** Marwa M. Abu-Serie, Noha H. Habashy, Wafaa E. Attia

**Affiliations:** 1 Medical Biotechnology Department, Genetic Engineering and Biotechnology Research Institute, City for Scientific Research and Technology Applications (SRTA-City), New Borg El Arab, Alexandria, 2934 Egypt; 2 Department of Biochemistry, Faculty of Science, Alexandria University, Alexandria, 21511 Egypt

**Keywords:** Phytochemistry, Antioxidant, Anti-inflammatory, Malaysian *Ganoderma lucidum*, Egyptian *Chlorella vulgaris*, synergism

## Abstract

**Background:**

Since oxidative stress and inflammation are two linked factors in the pathogenesis of several human diseases. Thus identification of effective treatment is of great importance. Edible mushroom and microalgae are rich in the effective antioxidant phytochemicals. Hence, their beneficial effects on oxidative stress-associated inflammation are extremely required to be investigated.

**Methods:**

This study evaluated the functional constituents, antioxidant and anti-inflammatory activities of Malaysian *Ganoderma lucidum* aqueous extract (GLE) and Egyptian *Chlorella vulgaris* ethanolic extract (CVE). Also, the synergistic, addictive or antagonistic activities of the combination between the two extracts (GLE-CVE) were studied. Expression of inducible nitric oxide synthase, cyclooxygenase-2, and nuclear factor-kappa B, as well as levels of nitric oxide, tumor necrosis factor (TNF)-α, lipid peroxidation, reduced glutathione and antioxidant enzymes were determined using in vitro model of lipopolysaccharide-stimulated white blood cells.

## Results & discussion

Different phytochemical compounds and appreciable amounts of minerals were detected in the extracts. GLE-CVE showed higher total antioxidant capacity and anti-radical effects than individual extracts. In addition, it attenuated the lipopolysaccharide-induced inflammation and oxidative stress in stimulated white blood cells synergistically. This occurred through downregulating the inflammatory mediators (TNF-α, and expression of inducible nitric oxide synthase, cyclooxygenase-2, and nuclear factor-kappa B) and enhancing the cellular antioxidant indices. Subsequently these led to suppression the cellular elevation of nitric oxide and lipid peroxidation. Moreover, this anti-inflammatory-dependent antioxidant effect of the combined extract was significantly higher than standard anti-inflammatory drug (dexamethasone).

## Conclusion

Mixing of Malaysian GL and Egyptian CV was better in the most tested parameters than each separate extract and dexamethasone. This combined extract exerted highly synergistic antioxidant and anti-inflammatory activities, so it may represent a promising alternative treatment for the oxidative stress- and inflammation-mediated diseases.

## Background

Oxidative stress and inflammation are two linked pathophysiological processes; stimulated by abnormal production of free radicals and proinflammatory mediators causing extreme cellular damage which involves in the pathogenesis of human chronic diseases. One of which stimulators (reactive radicals and proinflammatory mediators) can be easily induced by another. Reactive oxygen and nitrogen species (RONS) can induce intracellular signaling cascade to stimulate expression of proinflammatory genes. On the other hand, the inflammatory mediators such as nuclear factor-kappa (NF-к) B, inducible nitric oxide synthase (iNOS), cyclooxygenase (COX)-2 and tumor necrosis factor (TNF)-α leading to excessive oxidative damage [1]. This oxidative stress-associated inflammation can be neutralized and detoxify by the antioxidant system like superoxide dismutase (SOD), glutathione peroxidase (GPX) and glutathione reductase (GR) as well as nonenzymatic molecules such as reduced glutathione (GSH) [2]. Many foods such as algae, and mushrooms are rich in antioxidant so, they could be used in the treatment of several diseases [3, 4].

Microalgae are ancient microorganisms; present on earth since 3.4 billion years ago and some of them considered as important human and animal food source. *Chlorella vulgaris* (CV) is unicellular green eukaryotic microalga, grows in fresh water and belongs to Chlorellaceae family. It can be used as energy source and food supplement due to its richness in proteins, lipids, carbohydrates, pigments, and vitamins. In addition, CV has important pharmacological potentials because it shows anti-cancer and immune-modulating effects, lowers the risk of atherosclerosis and supports protection from age-related diseases. Therefore, it is very important for human health and as a result, it was taken in different forms such as tablets, capsules, powder, and extracts [5].

Many mushrooms have important pharmacological effects such as *Ganoderma lucidum* (GL), which belongs to Ganodermaceae family of polyporales. It is cultivated in Taiwan, China, Japan, Korea, North America and Malaysia and is known as wood-decaying fungus [6]. The GL is considered as a medicinal mushroom that can be used in the treatment of coronary heart diseases, nephritis, hepatitis, asthma, and arthritis [7].

The present study investigated for the first time, the constituents, antioxidant and anti-inflammatory potentials of the Malaysian GL hot water extract (GLE). Also, this work evaluated, for the first time, the antioxidant and anti-inflammatory activities of mixing CV ethanolic extract (CVE) with GLE. Hence, the combination of different foods may affect their properties so, it is important to evaluate the effect of their mixing. This combination may provide certain effect greater (synergistic), similar (additive), or less (antagonistic) than the sum of the single components effects. The observed value (OV) of the combined extracts is compared with the expected value (EV), which is the sum of the half values of the individual extracts under test. If significantly the OV showed enhancing effect more than the EV, the combined extract exerted synergistic interaction [8], while if the opposite happened, the interaction was antagonistic. The additive interaction was obtained if there was no significant difference between the two values. These different effects depending on the types and concentration of the compounds in each food and the interaction or co-existence of these compounds.

## Materials and methods

### Chemicals

Folin-Ciocalteau reagent, gallic acid (GA), ursolic acid (UA), rutin (RU), catechin, dextran sulfate (DS), 2,2 diphenyl-1-picrylhydrazyl (DPPH), Griess reagent, thiobarbituric acid (TBA), riboflavin, nitrobluetetrazolium (NBT), toluidine blue, lipopolysaccharide (LPS, from *Escherichia coli*), GSH and dexamethasone (Dex) were obtained from Sigma-Aldrich (St. Louis, MO, USA). Ascorbic acid (Asc) was supplied from Riedel-de Haën, Germany. RPMI-1640 medium and fetal bovine serum (FBS) were purchased from Lonza (USA).Gene JET RNA purification kit and cDNA synthesis kit, 2X SYBR green master mix kit and protease inhibitor cocktail were obtained from Thermo Scientific, USA. Human TNF-α ELISA kit was obtained from Ray Biotech, USA. Primers for NF-кB, iNOS, COX-2 and were purchased from Bioneer, Korea. Other chemicals were obtained with high grade.

### Extraction methods

The GL powder was obtained from DXN pharmaceutical SDN BHD (410692-K, Lot 1109, Malaysia). For GLE preparation; 12 g of GL powder (7.2 g mycelium and 4.8 g fruit body) were subjected twice to 200 mL of hot water at 70 °C for 2 h. The extract was filtered, then freeze-dried by the lyophilizer (Telstar, Terrassa, Spain) and the powder (GLE, yield of 46%) was kept at − 20 °C prior to use. CV was obtained from the Algal Biotechnology Unit, National Research Centre, Dokki, Giza, Egypt in the freeze-dried form. Cells were disrupted by liquid nitrogen, extracted using ethanol and then centrifuged at 940 xg for 20 min. The supernatant was freeze- dried to obtain the powdered extract (CVE, yield of 2.5%) which was stored at − 20 °C until used.

### Synergy testing

To study the possible antioxidant and anti-inflammatory synergistic effects, an equal volume of CVE (63.5 μg/mL) was mixed with GLE (4.1 μg/mL) “GLE-CVE”. These indicated concentrations of extracts are equivalent to their estimated safe concentrations toward human white blood cells (WBCs, the cells that used in the cytotoxicity assay).

### Phytochemical analyses

Total phenolics content was quantified as GA equivalents in mg/g extract using Folin-Ciocalteau reagent [9]. Total flavonoids were determined colorimetrically according to Zhishen et al. 1999 [10].Total triterpenoids were assessed using vanillin and UA as standard [11]. Total tannin content was determined spectrophotometrically using the standard curve of catechin [12]. Furthermore, sulfated polysaccharides (SPs) were determined using toluidine blue reagent and DS standard [13].

### Minerals estimation

Analysis of minerals (Cu, Se, Zn, Fe, Ca, and Mg) in each extract was done by using atomic absorption spectrophotometer (Perkin Elmer model 2380, Norwalk, CT, USA). According to Hack method [14], 1 g of the extract was added to 5 mL of 55% nitric acid and 2 mL of 70% perchloric acid and digested by heating at 100 °C for 1 h. After cooling, the digested solution was filtered and diluted 10 times, then an aliquot of this solution was used for the study of the minerals.

### HPLC analysis for identification and quantification of phenolics

The extract (20 μL) was separated on 150 mm × 4.6 mm, 5 μm Eclipse XDB–C18 column (Agilent Technologies, Palo Alto, CA, USA) using 1% formic acid: acetonitrile: 2-propanol (70:22:8) as a mobile phase. The separation was performed at 320 nm, pH 2.5 and flow rate of 0.75 mL/min [15].

### Determination of the antioxidant activity

The antioxidant activity of the single (GLE and CVE) and combined extract (GLE-CVE) were evaluated using more than one antioxidant method to give a comprehensive prediction of the antioxidant potentials. Total antioxidant capacity (TAC), radical scavenging, ferric reducing and anti-lipid peroxidation activities methods were examined.

### The TAC assay

Briefly, 100 μL of each extract (single or combined) or serial concentration of Asc (0–1 mg/mL) was added to 1.9 mL of the reagent solution (0.6 M H_2_SO_4_, 28 mM sodium phosphate and 4 mM ammonium molybdate). The mixtures were incubated in an oven at 95 °C for 90 min; then the absorbance was measured at 695 nm. TAC was expressed as Asc equivalents in mg/g of the extract [16].

### Radical scavenging activity

The present study evaluated the ability of GLE, CVE, and GLE-CVE to scavenge NO, DPPH, superoxide anion, and hydroxyl radicals. NO scavenging activity was measured using sodium nitroprusside and Griess reagent. This reaction produced colored azo dye with a maximum absorbance at 490 nm [17]. The DPPH scavenging activity was determined by recording the absorbance of the non-scavenged DPPH at 490 nm [18]. Superoxide anion radical scavenging ability of extract was evaluated by the inhibiting superoxide anion-mediates reduction of yellow NBT (1.5 mM) to purple formazan and the decreasing in absorbance at 530 nm [19]. While the hydroxyl radical scavenging activity was determined using the salicylic acid method and the absorbance was measured at 510 nm [20]. A relationship between % of radical scavenging and Log concentrations of each extract was plotted for calculating the IC_50_ value (50% inhibitory concentration) for each radical and the results were compared with Asc.

### Ferric reducing power

Ferric reducing power was determined using potassium ferricyanide-ferric chloride method [21]. Serial concentrations of each extract (single or combined) or Asc were mixed separately with 2.5 mL of 0.2 M phosphate buffer (pH 6.6) and 2.5 mL 1% potassium ferricyanide. The mixtures were incubated for 20 min at 50 °C then 2.5 mL of 10% trichloroacetic acid (TCA) was added. After that, 2.5 mL of the mixture was mixed with equal volume of distilled water and 0.5 mL of ferric chloride (1%) then the solution was incubated at 25 °C for 30 min and the absorbance was read at 700 nm. The EC_50_ value (effective concentration of the extract or Asc that reduce iron by 50%) was calculated from the relation between % of iron reduction vs. Log concentration of the extract or Asc.

### Anti-lipid peroxidation assay

The degree of lipid peroxidation was assessed by determination of the thiobarbituric acid reactive substances (TBARS) level [22] using the rat liver homogenate. Three healthy Albino rats (90–95 g) were obtained from MISR University for Science and Technology, Egypt (animal welfare assurance no. A5865–01), and handled according to the national guide for the care and use of laboratory animal. The animals were sacrificed to obtain the liver, which was used in the preparation of 10% homogenate with cold phosphate buffered saline (PBS). The homogenate was centrifuged at 940 xg for 5 min, then 1 mL of the supernatant was added to equal volume of each concentration of the single or combined extract or Asc. Then the lipid peroxidation was initiated by adding 100 μL of 15 mM FeSO_4_ solution, and the mixture was incubated for 30 min. One hundred microliters of this mixture were incubated for 10 min with 1.5 mL of 10% TCA and the mixture was centrifuged at 940 xg. The supernatant was mixed with 1.5 mL of 0.67% TBA; then heated for 30 min in boiling water bath and the intensity of the pink color was measured at 535 nm.

### Cytotoxicity and anti-inflammatory assays

The cytotoxicity and anti-inflammatory activities of GLE, CVE and GLE-CVE were determined using human WBCs. The blood was collected from ten healthy volunteers in heparin tubes and mixed gradually with a fresh cold lysing solution (80.2 mg % ammonium chloride, 8.4 mg % NaHCO_3_, and 3.7 mg % EDTA). Then it was centrifuged at 1650 rpm for 5 min and the pellet (WBCs) was washed twice with RPMI-1640 medium. Then cells were stained by trypan blue for checking the viability and counting using a phase contrast inverted microscope (Olympus, Tokyo, Japan). Finally, cells were cultured in RPMI-1640 medium containing 10% FBS for using in the cytotoxicity and anti-inflammatory assay.

### Cytotoxicity assay

About 100 μL of serial dilutions of single or combined extract or Dex were added to 1 × 10^5^ WBCs/well in 96-well plate and incubated in CO_2_ incubator (New Brunswick Scientific, Netherlands) at 37 °C, 5% CO_2_, and 90% relative humidity. After 72 h, 20 μL of MTT (5 mg/mL in PBS) was added to each well and incubated in CO_2_ incubator for further 3 h, and then centrifuged for 10 min at 280 xg. One hundred microliters of DMSO was added (formazan crystals, MTT byproduct) and the absorbance was read at 570 nm using ELISA reader (BMG Lab Tech, Germany) [23]. Cell viability was determined and a relation between the cell viability and the studied extract or Dex concentrations was plotted for calculating the safe concentrations (EC_100_, 100% cell viability).

### Anti-inflammatory assay

The WBCs (1 × 10^5^ cells) were seeded in each well of 96-well plate and mixed with equal volume of 1 mg/ml LPS for induction of inflammation. Cells were incubated in CO_2_ incubator for 24 h and then treated with 100 μL of medium or different dilutions of the safe concentration of extracts (single or combined) or Dex. The MTT solution was added to each well after 72 h incubation, and the method was completed as described above. The stimulation index (SI, the ratio between the absorbance of the extract or Dex-treated LPS-stimulated cells versus the absorbance of untreated cells) was estimated. Also, the effective concentration (EC), which is able to reverse the abnormal SI value of LPS-stimulated cells to the normal value (SI ≈ 1) was calculated.

### TNF-α, NO, and lipid peroxidation assays

In 96-well plate, 1 × 10^5^ WBCs/ well were stimulated by 1 mg/ml LPS for 24 h and then treated for 72 h with extracts (single or combined) or Dex at their EC. After centrifugation, 150 μL of supernatant per well was collected and used for determination of TNF-α, NO and lipid peroxidation levels. TNF-α was quantified following the manufacture’s protocol using ELISA kit and NO level was assessed by measurement of the nitrite using the Griess reaction [17]. While the lipid peroxidation level was determined by estimating the TBARS level [22].

### Quantitative real-time polymerase chain reaction (qRT-PCR) of some inflammatory mediators

About 1 × 10 ^6^ WBCs were seeded per well of 6-well plates; then were exposed to LPS for 24 h. After that, cells were treated for 72 h with EC of the extracts (single or combined) or Dex. Then total RNA was extracted from the untreated, LPS-stimulated, extract- or Dex plus LPS-treated WBCs following the manual protocol of Gene JET RNA purification kit. One microgram of each RNA sample was used for cDNA synthesis by reverse transcriptase-PCR using the cDNA synthesis kit. Levels of gene expressions of target genes and β-actin (reference gene) were quantified by RT-PCR (Qiagen, Germany) using the gene-specific forward and reverse primers. The following primers were used: NF-кB, forward: 5′-ATGGCTTCTATGAGGCTGAG-3, reverse: 5′-GTTGTTGTTGGTCTGGATGC-3′; iNOS, forward: 5′-GTTCTCAAGGCACAGG TCTC-3′, reverse: 5′-GCAGGTCACTTATGTCACTTATC-3′; COX-2, forward: 5′-ATCATTCACCAGGCAAATTGC-3′, reverse: 5′-GGCTTCAGCATAAAGCGTTTG-3′; and β-actin, forward: 5′-AAGCAGGAGTATGACGAGTCCG-3′, reverse: 5′-GCCTTCATACATCTCAAGTTGG-3′.

The reaction mixture contained 0.3 μL of 10 μM forward primer, 0.3 μL of 10 μM reverse primer, 12.5 μL of 2X SYBR green master mix, and 50 ng cDNA template. Then, the total volume was completed with nuclease-free water to 25 μL. The qPCR program was applied as one cycle of enzyme activation at 95 °C for 15 min followed by 40 cycles of denaturation at 95 °C for 15 s, annealing at 60 °C for 1 min and extension at 72 °C for 30 s. The expression of target genes was calculated using the comparative Ct method (threshold cycle number at cross-point between amplification plot and threshold). The CT values of each target gene were normalized to that of β-actin according to manufacturer’s instructions and the change in expression (2^−ΔΔCT^) was calculated.

### Cellular antioxidant indices

One million WBCs were seeded per well in 6-well plate, stimulated with LPS and treated with the extracts (single or combined) and Dex as explained in the previous section. The untreated and treated cells were lysed in PBS-containing protease inhibitor cocktail using sonicator in an ice bath*,* and then the cell lysate was used in the determination of the antioxidant indices. The level of GSH was determined by generating a yellow-colored product with Ellman’s reagent and absorbance was measured at 412 nm [24]. The GPX activity was determined using GSH and cumene hydroperoxide as substrates. The total and unreacted GSH were measured, after reacting with Ellman’s reagent, at 412 nm for calculating the consumed GSH and subsequently GPX activity [25]. The GR activity was measured at 340 nm following the decrease in absorbance that induced by oxidized glutathione in the presence of NADPH [26]. The activity of Cu/Zn SOD was assessed using pyrogallol autooxidation method [27], in which the change in absorbance during 2 min was measured at 420 nm. The unit of activity is defined as the amount of enzyme that inhibits the rate of autooxidation of 20 mM pyrogallol by 50% under standard conditions. Protein content was quantified for the estimation specific activity of each above- mentioned enzymes (U/mg protein) using Bradford Coomassie brilliant blue assay [28].

### Data analysis

The experimental results were expressed as a mean ± standard error (S.E.) of three measurements and were analyzed by SPSS version 16. The mean values were compared using one-way analysis of variance (ANOVA) by Duncan’s test and significance was determined at *p* < 0.05. The OV and EV were compared using independent-samples t-test. IC_50_, EC_50_ and EC_100_ values with appropriate 95% confidence interval based on non-linear regression were calculated using GraphPad PRISM software version 6 by fitting a sigmoidal dose-response curve (variable slope).

## Results and discussion

### Phytochemical and minerals contents

**Fig. 1:**
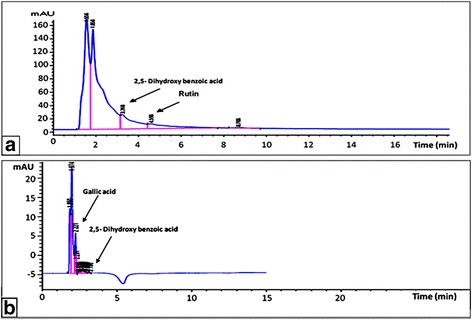
HPLC chromatograms of the phenolic compounds in (a) GLE, and (b) CVE

Table 1 shows the concentrations of the functional constituents (phytochemicals and minerals) of GLE and CVE. Different types of phenolic compounds were detected in GLE and CVE by HPLC analysis. Chlorogenic, caffeic, tannic, 3,4-dicaffeoyl quinic, 3,5-dicaffeoyl quinic, 4,5-dicaffeoyl quinic, cinnamic and 2,5-dihydroxy benzoic acids, GA, quercetin, RU, catechin, and phloridzin were used as standard phenolics. The chromatogram profiles (Fig. 1) show the presence of 0.027 ± 0.023 mg of 2,5-dihydroxybenzoic acid and 0.028 ± 0.050 mg of RU per gram of GLE while one gram of CVE contains 0.36 ± 0.02 mg of 2,5 dihydroxybenzoic acid and 0.16 ± 0.01 mg of GA. The presence of these constituents in the studied extracts increases their quality and importance.

**Table 1:** Phytochemicals and minerals composition of the studied extracts

Constituents	*Ganodermalucidum* extract (GLE)	*Chlorella vulgaris* extract (CVE)
Phytochemicals (mg/g extract)		
Total phenolics	4.44 ± 0.08^**a**^	5.18 ± 0.58^**a**^
Flavonoids	0.14 ± 0.00^**b**^	3.46 ± 0.21^**a**^
Tannins	1.24 ± 0.00^**a**^	1.02 ± 0.01^**a**^
Triterpenoids	23.88 ± 0.00^**a**^	5.45 ± 0.25^**b**^
SPs	3.66 ± 0.03^**a**^	1.04 ± 0.05^**b**^
Minerals (μg/g extract)		
Cu	6.4 ± 0.00^**b**^	30.08 ± 0.01^**a**^
Se	606.7 ± 0.00^**a**^	276 ± 0.00^**b**^
Zn	16.3 ± 0.00^**b**^	4300 ± 0.01^**a**^
Fe	1100 ± 0.00^**b**^	4180 ± 0.00^**a**^
Ca	00.00	20,000 ± 0.00
Mg	00.00	4260 ± 0.01

Results are expressed as Mean ± S.E. Different letters are significantly different within the same row at *p* < 0.05

It is well-known that phytochemicals and minerals are essential for human health and have many biological functions, especially in the antioxidant defense system. Since many phytochemical compounds such as terpenoids, phenolics, vitamins, tannins, carotenoids, and polysaccharides are considered as highly effective antioxidants [29]. In addition, certain trace elements (Cu and Zn, Se, and Fe) are vital for the activities of several antioxidant metalloenzymes (SOD, GPX, and catalase, respectively) [30, 31]. Therefore, the presence of these constituents in GLE and CVE increase their importance in human nutrition health.

### Antioxidant activities of single and combined extracts

**Fig. 2:**
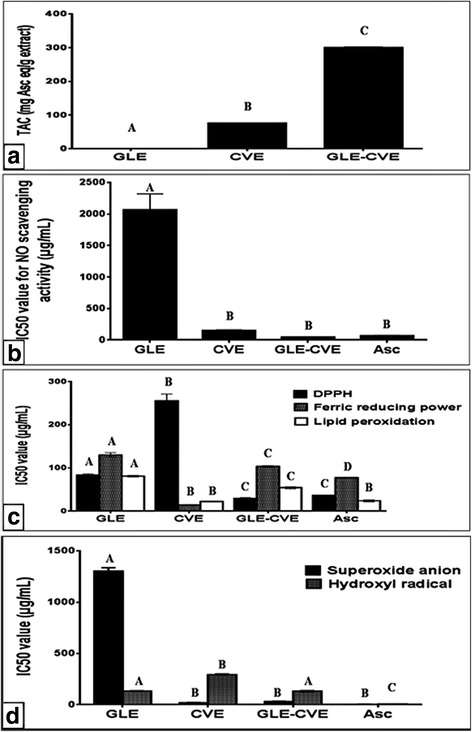
In vitro antioxidant activities of GLE, CVE, and GLE-CVE combination in comparison with Asc. (a) Total antioxidant capacities (TAC) (b) NO scavenging activity (c) superoxide anion and hydroxyl radical scavenging activities, and (d) DPPH scavenging activity, anti-lipid peroxidation effect, and ferric reducing power. Results are expressed as Mean ± S.E. Different letters for the same parameter are significantly different at *p* < 0.05

The graphs presented in Fig. 2 reveal the antioxidant activities of the single (GLE and CVE) and combined (GLE-CVE) extracts. The TAC results (Fig. 2a) showed that CVE had significantly (*p* < 0.05) higher activity than GLE. Due to the TAC assay reflects the functional constituents of the extract so, the higher TAC of CVE may be attributed to its phytochemical contents. The higher TAC of CVE reinforced it to be better scavenger for the free radicals than GLE. Since CVE had IC_50_ values for NO and superoxide anion radical nonsignificant different with that of the Asc and significantly higher values for DPPH and hydroxyl radicals. Meanwhile, the IC_50_ values of GLE for all the examined radicals were significantly higher than Asc values (Fig. 2b-d). These results meant that CVE had potent scavenging effects for NO and superoxide anion radicals and weak abilities for other radicals, while GLE had the lowest scavenging activities for all of these radicals as compared with CVE and Asc. This may be owed to certain phytochemical compounds in CVE such as carotenoids (terpenoids), which are responsible for the TAC and the superoxide anion radical scavenging effect of CV [32, 33].

Considering the synergism (Table 2), GLE-CVE exhibited highly TAC (OV > EV) and subsequently, this led to enhancement of its anti-radical activities at lower IC_50_ values (OV < EV) than single extracts indicating the high synergistic effect. Comparing with Asc, the combined extract exhibited similar TAC and anti-radical potency except for the hydroxyl radical scavenging activity that revealed significantly lower potency. These results elucidated that mixing strong and weak antioxidants together may improve the overall antioxidant ability of the combined extract. The synergism effects of GLE-CVE may be owed to the interaction between the endogenous phytochemicals in it. Since the synergistic interactions of triterpenes/phenolics [34] and carotenoids/phenolics [35] were observed previously. While the exact phytochemicals interactions which may be occurred after mixing CVE with GLE need to further analyses and investigations.

**Table 2:** The observed and expected values of antioxidant and anti-inflammatory activities of the combined extract

In vitro antioxidant models											
		TAC (mg Asc eq/ g extract)	Effect	NO scavenging activity (IC_50_, μg/mL)	Effect	DPPH scavenging activity (IC_50_, μg/mL)	Effect	Reducing power (IC_50_, μg/mL)	Effect	Superoxide scavenging activity (IC_50_, μg/mL)	Effect
GLE-CVE	**OV**	**301.00 ± 1.00** ^**a**^	Sy	**46.12 ± 2.92** ^**a**^	Sy	**29.48 ± 1.45** ^**a**^	Sy	**103.47 ± 2.04** ^**a**^	An	**30.01 ± 3.45** ^**a**^	Sy
	EV	38.83 ± 2.16^**b**^		1111.02 ± 128.75^**b**^		169.29 ± 9.36^**b**^		71.98 ± 1.99^**b**^		660.73 ± 14.92^**b**^	
		Hydroxyl radical scavenging activity (IC_50_, μg/mL)			Effect	Anti-Lipid peroxidation activity (IC_50_, μg/mL)					Effect
	**OV**	**131.6 ± 5.42** ^**a**^			Sy	**54.15 ± 2.45** ^**a**^					Ad
	EV	213.18 ± 2.13^**b**^				51.49 ± 0.96^**a**^					
Inflammatory mediators											
		NO (nmol/mL)	Effect	iNOS (Expression)	Effect	COX-2 (Expression)	Effect	NF-кB (Expression)	Effect	TNF-α (mmol/mL)	Effect
GLE-CVE	**OV**	**39.26 ± 4.37** ^**a**^	Sy	**0.146 ± 0.006** ^**a**^	Sy	**0.056 ± 0.001** ^**a**^	Sy	**0.49 ± 0.015** ^**a**^	Sy	**242.88 ± 18.5** ^**a**^	Sy
	EV	52.08 ± 1.08^**b**^		1.29 ± 0.29^**b**^		0.18 ± 0.002^**b**^		0.85 ± 0.038^**b**^		422.04 ± 2.65^**b**^	
Oxidative stress parameters											
		Lipid peroxidation (nmoL/mL)	Effect	GSH (nmoL/mL)	Effect	Superoxide dismutase (SOD, IU/mg protein)	Effect	Glutathione peroxidase (GPX, IU/mg protein)	Effect	Glutathione reductase (GR, IU/mg protein)	Effect
GLE-CVE	**OV**	**7.29 ± 0.84** ^**a**^	Sy	**0.59 ± 0.01** ^**a**^	Sy	**0.66 ± 0.0002** ^**a**^	Sy	**0.07 ± 0.001** ^**a**^	Sy	**3.25 ± 0.06** ^**a**^	Ad
	EV	59.58 ± 1.62^**b**^		0.48 ± 0.0002^**b**^		0.63 ± 0.002^**b**^		0.06 ± 0.0001^**b**^		3.19 ± 0.02^**a**^	

Data are expressed as Mean ± S.E. GLE-CVE; combined extractof Malaysian *Ganodermalucidum* and Egyptian *Chlorella vulgaris* extracts, OV; observed value, EV; expected value, Sy; synergistic effect, Ad; additive effect, An; antagonistic effect. Different letters between each OV and EV within the same column are significantly different at *p* < 0.05

The ferric reducing power method was investigated the reducing capacity of the tested extract to convert ferricyanide (Fe^+ 3^) to ferrocyanide (Fe^+ 2^). Fig. 2c demonstrates that GLE had the weakest ferric reducing power, whereas CVE exhibited the strongest reductive effect in comparison with the Asc. In addition, CVE had higher anti-lipid peroxidation activity than GLE and as potent as Asc (Fig. 2c). The better reductive effect of CVE probably related to its large TAC that was higher than that of GLE (Fig. 2a). Also, the high TAC and scavenging potential for the superoxide anion radical may interpret the ability of CVE to inhibit the FeSO_4_-induced lipid peroxidation (Fig. 2c). Because Fe^+ 2^ may initiate lipid peroxidation through its oxidation by the molecular oxygen to Fe^+ 3^ producing superoxide radicals that abstract the methylene carbon-hydrogen atom from the polyunsaturated fatty acid side chain [36].

On the other hand, the addition of CVE to GLE produced an antagonistic reductive effect (OV > EV, Table 2) that was observed by increasing IC_50_ values and additive anti-lipid peroxidation (OV ≈ EV) effects with potency less than Asc (Fig. 2c). The decrease in the reductive power of GLE-CVE may be related to the interaction between certain types of its phytochemicals and this may lead to non-improvement of its anti-lipid peroxidation activity.

### Effect of the single and combined extracts on WBCs viability

The results revealed that the safe concentrations (EC_100_, 100% viability) for the extracts on WBCs were decreased in the order CVE > GLE-CVE > Dex > GLE. The values of EC_100_ (μg/mL) for these extracts were 63.53 ± 3.15, 40.83 ± 2.23, 26.39 ± 0.60 and 4.13 ± 1.59, respectively. All of these values are significantly (*p* < 0.05) different from each other.

### Induction of inflammation in WBCs by LPS

**Fig. 3:**
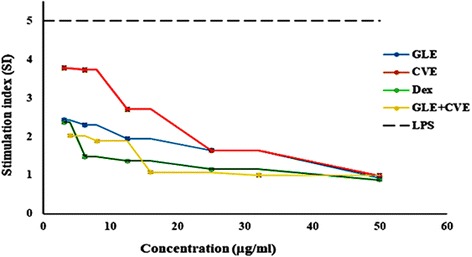
Effect of serial concentration of GLE, CVE, GLE-CVE combination, and Dex on the abnormal proliferation of LPS-exposed WBCs in the term of SI. Results are expressed as Mean ± S.E

**Fig. 4:**
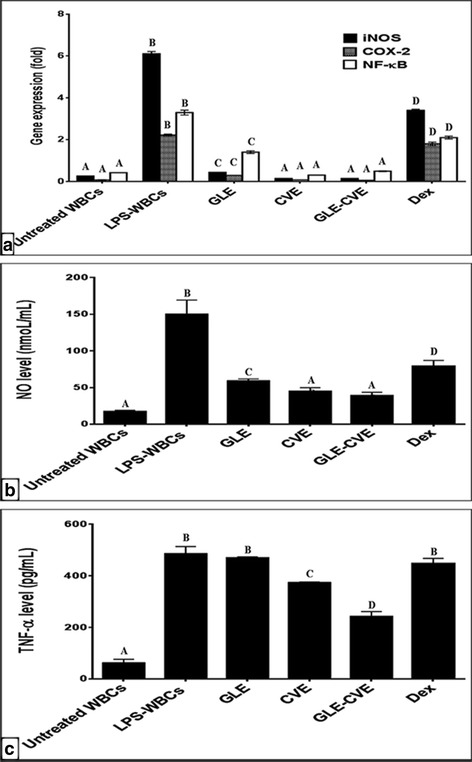
Effect of GLE, CVE, GLE-CVE combination, and Dex on LPS-induced inflammation in WBCs. (a) the change in gene expression of iNOS, COX-2, and NF-кB (b) NO level (c) the protein level of TNF-α. Results are expressed as Mean ± S.E. Different letters for the same parameter are significantly different at *p* < 0.05

The results demonstrated that incubation of human WBCs with LPS abnormally stimulated their proliferation as it was declared by the highest SI > 5 (Fig. 3) and upregulated the expression of NF-κB, iNOS and COX-2 by 3.3, 2.2 and 6.1 folds, respectively (Fig. 4a) versus control untreated WBCs. In addition, NO (Fig. 4b) and TNF-α (Fig. 4c) levels were significantly elevated in LPS-exposed WBCs (LPS-WBCs) by 753.7% and 683.60%, respectively.

Lipopolysaccharide (endotoxin) is the important component in the outer membrane of Gram-negative bacteria; it consists of large polysaccharide, core oligosaccharide, and lipid-A region. This latter is responsible for most of LPS toxicity and it is a highly conserved part of it. LPS is a potent inflammatory inducer due to its ability to stimulate abnormal proliferation of leukocytes which is used as a marker of inflammation [37] and to activate the NF-кB pathway, which regulates the expression of the pro-inflammatory mediators (iNOS, COX-2, and TNF-α) to boost the inflammatory response [38].

### Suppression of LPS-induced inflammation by single and combined extracts

All of the studied single and combined extracts, as well as Dex, were able to decrease the abnormal elevation in the proliferation of LPS-exposed WBCs in a dose-dependent manner (Fig. 3). The EC values of GLE, CVE, GLE-CVE and the standard anti-inflammatory drug (Dex) that able to reverse the abnormal stimulation (SI > 5) in the LPS-exposed WBCs to the normal (SI = 1) were 38.5 ± 0.2, 37.7 ± 4.6, 13.6 ± 0.4 and 30.4 ± 0.3 μg/mL, respectively. These EC values were significantly different from each other except for GLE and CVE.

The treatment of LPS-WBCs with the ECs of the studied single and combined extracts was able to reverse the LPS-induced inflammation by downregulating the production of the inflammatory mediators (Fig. 4). This action gave the studied extracts (single and combined) a great importance due to these key mediators involve in various chronic diseases such as asthma and cancer. GLE and CVE were able to downregulate NF-кB expression by 57.4% and 90.8%, respectively as compared with LPS-stimulated WBCs (Fig. 4a). Their inhibition of NF-кB pathway resulted in significant decreases in the expression fold of iNOS by 92.81% and 97.59%, respectively and COX-2 by 87.10% and 96.49%, respectively (Fig. 4a). Moreover, CVE significantly reduced the level of NO (Fig. 4b) and TNF-α (Fig. 4c) by 70.01% and 22.99%, respectively. While GLE significantly suppressed the NO level (61.07%) and nonsignificantly decreased the TNF-α protein (3.18%) comparing to LPs-stimulated WBCs. In agreement with these results, the previous studies reported the anti-inflammatory activities of Korean GL, and Indian CV in LPS-stimulated macrophages [39, 40]. Dex also suppressed the level of NF-кB, iNOS, COX-2, NO and TNF-α by 36.8%, 45.10%, 19.41%, 47.36% and 7.80%, respectively. These results revealed that the tested single extracts had more effective anti-inflammatory activities than Dex. Furthermore, the studied extracts were able to normalize some of these mediators and returned them to the values of the untreated WBCs. Since CVE normalized the gene expression of NF-кB, iNOS and COX-2 and GLE normalized iNOS and COX-2 expression. The anti-inflammatory activities of the studied extracts may be related to their active phytoconstituents. Hence, certain phenolics such as RU and GA are potent inhibitors for NF-кB, iNOS, COX-2, NO and TNF-α in LPS-stimulated macrophages [41, 42]. In addition, other components in the studied extracts such as triterpenoids [43] and SPs [44] were able to inhibit the LPS-triggered expression of the inflammatory mediators.

The synergy test revealed that GLE-CVE caused significant attenuation for the expression of NF-кB, iNOS and COX-2 in the treated LPS-WBCs by 84.98%, 97.6% and 97.4%, respectively (Fig. 4a). In addition, it significantly reduced the levels of NO and TNF-α protein by 73.8% and 49.98%, respectively (Fig. 4b, c). These results indicated that GLE-CVE had a potent anti-inflammatory effect more than Dex. Furthermore, this combined extract not only downregulated the production of these inflammatory mediators but also returned nearly all of them to the normal values of untreated WBCs. After comparing the OVs and EVs (Table 2), the results demonstrated that GLE-CVE exerted highly synergistic (OV < < EV) inhibitory effect for all of the studied inflammatory mediators comparing with the single extracts. In accordance with these results, few studies were conducted to evaluate the synergistic anti-inflammatory activities of mixing GL with other foods [45].

### Induction of oxidative stress in WBCs by LPS

**Fig. 5:**
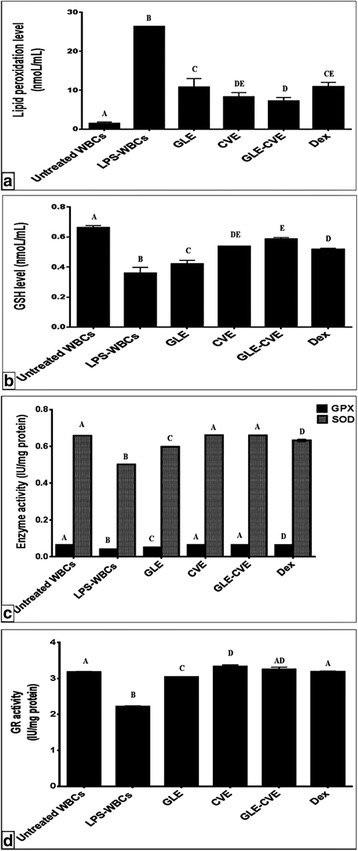
Effect of GLE, CVE, GLE-CVE, and Dex on LPS-induced oxidative stress in WBCs. (a) lipid peroxidation level (b) reduced glutathione (GSH) level (c) glutathione peroxidase (GPX) and superoxide dismutase (SOD) activities (d) glutathione reductase (GR) activity. Results are expressed as Mean ± S.E. Different letters for the same parameter are significantly different at *p* < 0.05

Fig. 5 demonstrates the ability of LPS to induce the oxidative stress on the WBCs through significant elevation of the lipid peroxidation level and significant reduction of the antioxidant indices versus the untreated cells. The level of GSH and the activities of SOD, GPX, and GR were significantly decreased by 45.67%, 23.69%, 36.58%, and 30.12%, respectively in LPS-WBCs as compared to the untreated cells. The current results agree with previous in vitro studies [46, 47].

LPS causes abnormal stimulation of the immune cells and enhances their secretion for ROS to eliminate the bacterial infection. ROS have an important role in the LPS toxicity through activating NF-кB pathway and mediating lipid peroxidation [48]. Thus the balance between free radicals and the antioxidant enzyme systems is important. SOD is the first line of antioxidant defense in the living cell due to its role in the dismutation of superoxide anion to H_2_O_2_ [27], which is detoxified by GPX in the presence of GSH. GSH is oxidized to GSSG [25] and then regenerated again from GSSG by GR to overcome the ROS damage [26]. The depletion in SOD, GPX and GR activities, which is concomitant with the high lipid peroxidation level in LPS-WBCs (Fig. 5a-d) indicated the inability of these cells to rid of free radicals. The accumulation of these ROS and other reactive species in LPS-WBCs can lead to inactivation or exhaustion of the antioxidant enzymes. Moreover, the decrease in the GSH level could lead to decrease the GPX activity and in turn decrease GSSG level, which resulted in depletion of the GR activity. Decreased GR activity can lead to a serious drop in GSH level, resulting in increased the lipid peroxidation and oxidative stress.

### Suppression of the LPS-induced oxidative stress in WBCs by the single and combined extracts

Fig. 5 illustrates that all of the studied extracts retained the balance between the antioxidants and ROS to overcome the LPS-induced oxidative stress. Therefore, the anti-inflammatory effect of these extracts may be mediated by suppression of the oxidative stress. Our results showed that GLE and CVE significantly reduced the cellular level of lipid peroxidation (Fig. 5a) by 58.1% and 68.4%, respectively as compared to LPS-WBCs and their potency was the same as Dex (58.8%). In addition, the results elucidated that GLE improved the GSH level and the activities of SOD, GPX, and GR by 17.14%, 19.10%, 23.51% and 36.83%, respectively comparing with LPS-WBCs (Fig. 5b-d). On the other hand, CVE enhanced these indices by 49.46%, 31.66%, 58.15% and 50.10%, respectively. The anti-lipid peroxidation effect of the studied extracts was probably due to the phenolic constituents of the extracts such as RU and gallic acid, which reduced the LPS-induced elevation of lipid peroxidation, NO and COX-2 activity [48, 49]. These results confirmed our in vitro antioxidant study for CVE, which interpreted its anti-radical effects and its ability to inhibit the lipid peroxidation (Fig. 2). Furthermore, the enhancement of the antioxidant indices by the studied extracts may be owed to their contents of certain polyphenolic compounds [48, 49] and SPs [50]. Moreover, the elevation in GSH level may be related to the elevation in GR activity, which catalyzes its regeneration from the oxidized form and this could be resulted also in enhancing the GPX activity. On the other hand, the data of the present study demonstrated the presence of considerable quantities of important minerals in the studied extracts (Table 1). The presence of these minerals, in particular, Cu, Zn, and Se considered as another cause for the activation of the antioxidant enzymes in the studied extract-treated cells. Since Cu and Zn are important elements for SOD catalytic activity and stability, respectively [31] and Se is essential for GPX activity and protein synthesis [30]. Inconsistency with these data, Indian GL [51], Taiwanian CV [52] and Malaysian CV [53] were reported to reduce the oxidative stress.

The GLE-CVE significantly suppressed the level of lipid peroxidation and improved the GSH level and the activities of the antioxidant enzymes (SOD, GPX, and GR) as compared with LPS-WBCs (Fig. 5). The percentage of these effects were 72.36%, 62.72%, 31.55%, 57.28% and 46.48%, respectively. The combined extract showed higher antioxidant potency than Dex beside its ability to normalize the activities of GPX and GR to be no significant difference from the control untreated WBCs. With respect to the synergism, GLE-CVE exhibited synergistic effects for all the antioxidant indices as compared with the single extracts except for GR, which showed additive effect (Table 2).

## Conclusions

In summary, the edible Malaysian GLE and Egyptian CVE are rich in natural antioxidants and anti-inflammatory phytochemical compounds. Also, they contain nutritionally valuable minerals like Cu, Se, Zn, and Fe. CVE showed higher antioxidant and anti-inflammatory activities than GLE in most of the studied parameters. The combination between CVE and GLE exhibited highly synergistic effects in most of the examined antioxidant and anti-inflammatory parameters. Most importantly, these activities of the combined extract were significantly higher than currently used anti-inflammatory drug (Dex), thus it may represent a promising alternative natural therapy. These findings could have important applications in the functional foods design and could play a crucial role in human therapeutics.

## Abbreviations

Ad: Additive effect
An: Antagonistic effect
COX-2: Cyclooxygenase-2
CVE: *Chlorella vulgaris* ethanolic extract
Dex: Dexamethasone
EC: Effective concentration
EV: Expected value
GA: Gallic acid
GLE: Malaysian *Ganoderma lucidum* hot water extract
GPX: Glutathione peroxidase
GR: Glutathione reductase
GSH: Reduced glutathione
IC_50_: 50% inhibitory concentration
iNOS: inducible nitric oxide synthase
LPS: Lipopolysaccharide
NF-кB: Nuclear factor-kappa B
OV: Observed value
RONS: Reactive oxygen and nitrogen species
RU: Rutin
SI: Stimulation index
SOD: Superoxide dismutase
SPs: Sulfated polysaccharides
Sy: Synergistic effect
TAC: Total antioxidant capacity
TBARS: Thiobarbituric acid reactive substances
TNF-α: Tumor necrosis factor-α
UA: Ursolic acid
